# Cross-Talk between Neurons and Immune Cells in Pruritus: from Mechanisms To Medicines

**DOI:** 10.1007/s11882-026-01255-8

**Published:** 2026-02-19

**Authors:** Nicole Khalil, Tomer Kagan, Gil Yosipovitch

**Affiliations:** 1 Miami Itch Center, Dr. Phillip Frost Department of Dermatology and Cutaneous Surgery, University of Miami Miller School of Medicine, Miami, FL USA; 2 Department of Physiology and Pharmacology, Gray Faculty of Medical and Health Sciences, Tel Aviv University, Tel Aviv, Israel; 3 Dr. Phillip Frost Department of Dermatology and Cutaneous Surgery & Miami Itch Center, University of Miami Miller School of Medicine, 5555 Ponce de Leon, Coral Gables, FL 33146 USA

**Keywords:** Immune system, Neural pathway, Chronic itch, Cytokines, Neuropeptides

## Abstract

**Purpose of Review:**

Chronic pruritus (CP) is among the most distressing symptoms and has a complex pathophysiology. This review aims to describe the mediators and mechanisms of neuroimmune crosstalk—a term referring the bidirectional interactions between the nervous and immune systems that underly itch pathogenesis and chronicity.

**Recent Findings:**

Type 2 cytokines (IL-4, IL-13, IL-31) directly activate pruriceptive neurons, resulting in itch sensation and nerve fiber sensitization via TRPV1/TRPA1, whereas keratinocyte-derived alarmins (TSLP and IL-33) amplify neural activation. Periostin, an emerging downstream mediator, binds integrin αVβ3 on neurons and induces macrophage IL-31 release, representing a link between immune and neural pathways. BNP, another emerging mediator that is co-expressed with IL-31, facilitates itch signaling in the spinal cord and induces keratinocyte production of itch mediators, highlighting its integral role in neuroimmune signaling.

**Summary:**

CP arises from a complex interplay between the immune system, sensory neurons, and keratinocytes. The success of therapeutic advances targeting cytokines, neuropeptides, and their receptors in recent years have confirmed the importance of understanding these complex underlying networks.

## Introduction

Chronic pruritus (CP) is defined as itch that lasts six weeks or longer. Numerous dermatologic, allergic, and systemic conditions are characterized by CP, which is a highly debilitating symptom with a lifetime prevalence of approximately 22% [1]. The pathophysiology underlying CP is complex and relies heavily on neuroimmune crosstalk—a term referring to bidirectional interactions between the nervous system and the immune system [2–4]. Immune cells, such as mast cells, eosinophils, basophils, T cells, macrophages and keratinocytes release mediators including cytokines, histamine, and neuropeptides which act on cutaneous sensory nerve fibers to cause itch sensation [4]. Activation or sensitization of sensory neurons by these mediators results in the release of neuromodulators that further stimulate immune cells, leading to a dysregulated, self-perpetuating cycle underlying the deleterious effects of CP on the skin [5]. Type 2 cytokines (e.g. IL-4, IL-13, and IL-31) in particular are well-known for their large role in amplifying itch sensation through both immune cell recruitment and sensitization of cutaneous sensory nerves [5, 6]. Evidence suggesting a role for other neuroimmune mediators- including periostin, brain natriuretic peptide (BNP), gastrin-releasing peptide (GRP), and others- has also begun emerging [7–9]. Understanding these mechanisms of crosstalk between the nervous and immune systems and identifying their key players is critical in developing effective and targeted treatments for CP. Although targeted treatments have significantly expanded in recent years with the development of several biologic medications, gaps in our understanding of the neuroimmune mechanisms underlying CP remain. This review aims to close some of these gaps to aid clinicians in diagnosing, monitoring, and treating patients with CP.

## Sensory Neurons and Itch Transmission

Sensory neurons play a pivotal role in itch transmission, integrating signals from the skin to the spinal cord and brain [4, 10]. Broadly, two major pathways are recognized: histaminergic and non-histaminergic itch [11].

Histaminergic itch is triggered by mast cell degranulation and release of histamine, which binds to histamine H1 and H4 receptors on mechano-insensitive C-fibers [12, 13]. This in turn activates intracellular cascades engaging Transient Receptor Potential Vanilloid 1 (TRPV1), a nonselective depolarizing ion channel [14, 15]. The wheal and flare response characteristic of urticaria underscores this mechanism, where antihistamines are therapeutically effective [4]. By contrast, most chronic pruritic disorders are mediated primarily by non-histaminergic pathways, and antihistamines have limited efficacy [4, 16].

Non-histaminergic itch dominates in chronic pruritic disorders such as atopic dermatitis (AD), prurigo nodularis (PN), chronic spontaneous urticaria (CSU), and chronic pruritus of unknown origin (CPUO) [16]. In these dermatoses, a diverse set of pruritogens including proteases, cytokines, alarmins and neuropeptides, activate G protein-coupled receptors (GPCRs) such as protease-activated receptors (PAR2/4) and Mas-related GPCRs (Mrgprs). For example, mouse MrgrpA3 and its human homolog MrgprX1 are activated by chloroquine and BAM8-22 peptides, while MrgprX2, expressed in both mast cells and neurons, has been implicated in AD skin, promoting neuroimmune cross talk [17]. Downstream, these receptors couple to Transient Receptor Potential Ankyrin 1 (TRPA1) channels, which serve as central ionic gateways for non-histaminergic itch [18]. In AD, TRPA1 expression is upregulated, and its inhibition reduces both scratching and inflammation. Similarly, PN shows evidence of robust activation of TRPA1 and partial activation of TRPV1 positive sensory fibers by cytokines such as IL-4, IL-13 and IL-31, explaining why these conditions respond better to targeted biologics or Janus kinase (JAK) inhibitors [19].

Neuropeptides released by sensory neurons further amplify these circuits. Substance P, acting on neurokinin-1 receptors and MrgprX2 on mast cells, promotes neurogenic inflammation and sustained itch [20]. Calcitonin gene-related peptide (CGRP), often co-released with Substance P, reinforces dorsal horn excitation [21]. BNP has emerged as a particularly important neuromodulator, linking neuronal and immune responses [22]. BNP is produced in dorsal root ganglion (DRG) neurons and acts on natriuretic peptide receptor A in the spinal cord to facilitate itch signaling. In AD, BNP expression is increased both in the epidermis and DRG, and it is often co-expressed with IL-31 receptors. IL-31 not only drives peripheral itch through TRPA1and TRPV1 positive fibers, but also induces BNP release, which in turn promotes keratinocyte production of pruritogenic cytokines and mediators [23]. This places BNP at the core of a feed-forward neuroimmune loop, distinguishing it from other neuropeptides and highlighting its potential as a therapeutic target.

Central processing of itch reflects the integration of these peripheral events. Pruriceptive C-fibers project to the DRG, where TRPV1 positive, TRPA1 positive and Mrgpr positive subsets transmit signals into the dorsal horn of the spinal cord. Within laminae I/II, interneurons and peptidergic pathways, particularly BNP and GRP positive neurons, activate GRP-receptor (GRPR) expressing neurons [24]. These spinal neurons constitute a critical itch-selective circuit, with inhibitory interneurons releasing neuropeptide Y or dynorphin to reduce excitability under normal conditions. Conversely, in chronic itch states such as AD and PN, inhibitory tone is reduced, leading to neural sensitization characterized by hyperknesis and alloknesis, which refer to heightened itch sensation from normally pruritic stimuli and itch sensation from non-pruritic stimuli, respectively [10, 25]. Furthermore, AD patients often experience itch and pain simultaneously due to blunting of normal pain-induced itch suppression [26]. From the dorsal horn, signals ascend via the spinothalamic and spinoparabrachial tracts to the thalamus, somatosensory cortex, insula, and limbic regions, where both the sensory discriminative and affective dimensions of itch are encoded. Functional imaging studies in AD corroborate this, showing cortical and subcortical activation correlating with disease severity and pruritus intensity [27].

## Immune Cells in Pruritus

Immune cells are therefore central to the initiation and maintenance of pruritus, working in conjunction with neurons and keratinocytes in a dynamic neuroimmune network. Among these, mast cells have historically been the most recognized, but other immune subsets such as basophils, eosinophils, macrophages, dendritic cells, T cells and fibroblasts contribute to the chronicity and severity of itch [10].

Mast cells are tissue-resident immune sentinels strategically positioned near cutaneous nerves. Their classical role in itch relies on IgE-mediated release of histamine, which activates H1 and H4 receptors on sensory neurons to trigger acute itch [13]. However, mast cells also release non-histaminergic mediators such as tryptase, leukotrienes, prostaglandins, and cytokines IL-4, IL-13 and IL-31 [23]. Tryptase acts on PAR2 and PAR4 on neurons and keratinocytes, while leukotrienes and prostaglandins amplify neuronal depolarization [18]. Mast cells therefore contribute not only to histamine-driven urticaria but also to chronic non-histaminergic itch. Importantly, they do not act in isolation. Crosstalk with basophils, eosinophils and dendritic cells creates a pro-pruritic immune environment. Basophils infiltrate chronic pruritic lesions and release IL-4, IL-13, IL-31 and leukotrienes that directly excite nerves [28]. Eosinophils release platelet-activating factor, substance P, IL-4, IL-13, and IL-31 while depositing cytotoxic proteins near nerve endings, sensitizing cutaneous neurons [29]. Dendritic cells, particularly myeloid and Langerhans cells, respond to keratinocyte alarmins such as thymic stromal lymphopoietin (TSLP) and IL-33 to promote Th2 polarization. These dendritic cells can also produce IL-31, correlating with itch severity [30].

T cells, especially Th2 subsets, are critical mediators of pruritic inflammation. IL-4 and IL-13, acting through IL-4Rα and JAK1, sensitize TRPV1 and TRPA1 positive sensory neurons and enhance their responsiveness to other pruritogens [31]. IL-31, produced mainly by Th2 but also by mast cells, eosinophils, basophils, and macrophages, directly excites DRG neurons via IL-31RA and oncostatin-M specific receptor subunit beta (OSMRβ), induces nerve fiber elongation, and promotes chronic itch [31]. OSM, mainly produced by dermal T cells and monocytes, also plays a role in sensitizing neurons via OSMRβ and has been found to be significantly increased in chronic itch conditions [32]. IL-33, an alarmin, further amplifies this cascade by stimulating Th2 cells, basophils, and type 2 innate lymphoid cells (ILC2s) to release type 2 cytokines, while directly exciting TRPA1 positive neurons through its receptor ST2. TSLP, produced by keratinocytes, promotes dendritic cell driven Th2 polarization and directly activates sensory neurons via TRPA1 [33]. These cytokines sustain a chronic loop of inflammation, barrier disruption and neuronal sensitization that perpetuates CP.

M2 macrophages play a key role in the pathogenesis and maintenance of itch in conditions such as AD and stasis dermatitis (SD) [34, 35]. M2 macrophages are highly concentrated in itchy skin of AD and SD patients and produce numerous pruritogenic molecules including IL-31, periostin, and several neurotrophic factors, which go on to promote itch and neural sensitization [34]. Furthermore, depletion of macrophages or blockade of IL-31 has been shown to reduce scratching in experimental models, highlighting the importance of interaction between these two mediators [35].

Keratinocytes are more than passive barriers, and act as amplifiers of itch. They produce TSLP, IL-33, periostin and nerve growth factor (NGF) in response to mechanical injury, protease signaling or cytokine exposure [23]. TSLP and IL-33 recruit and activate immune cells while simultaneously acting on sensory nerves. NGF promotes nerve elongation and branching, contributing to hyperinnervation in chronically pruritic skin [36]. Periostin, downstream of IL-4, IL-13 and TSLP, binds integrin αVβ3 and directly excites neurons via TRPV1 and TRPA1, while also inducing IL-31 production from macrophages [36]. Thus, keratinocytes amplify the feed-forward loop of CP.

Mast cell-neuron bidirectional loops exemplify neuroimmune crosstalk. Neuropeptides such as substance P and CGRP, released from cutaneous nerves during itch and scratching, activate mast cells via substance P receptor and MrgprX2 [20]. This triggers mast cell release of histamine, tryptase and IL-31, which in turn excites nerves and keratinocytes [23]. Substance P also induces keratinocyte NGF production, further fueling nerve growth and sensitization. BNP integrates into this loop by modulating both neuronal and immune responses, placing it at the crossroads of neuroimmune amplification [22].

## Synthesizing the Molecular Mechanisms of Neuroimmune Crosstalk

**Fig. 1:**
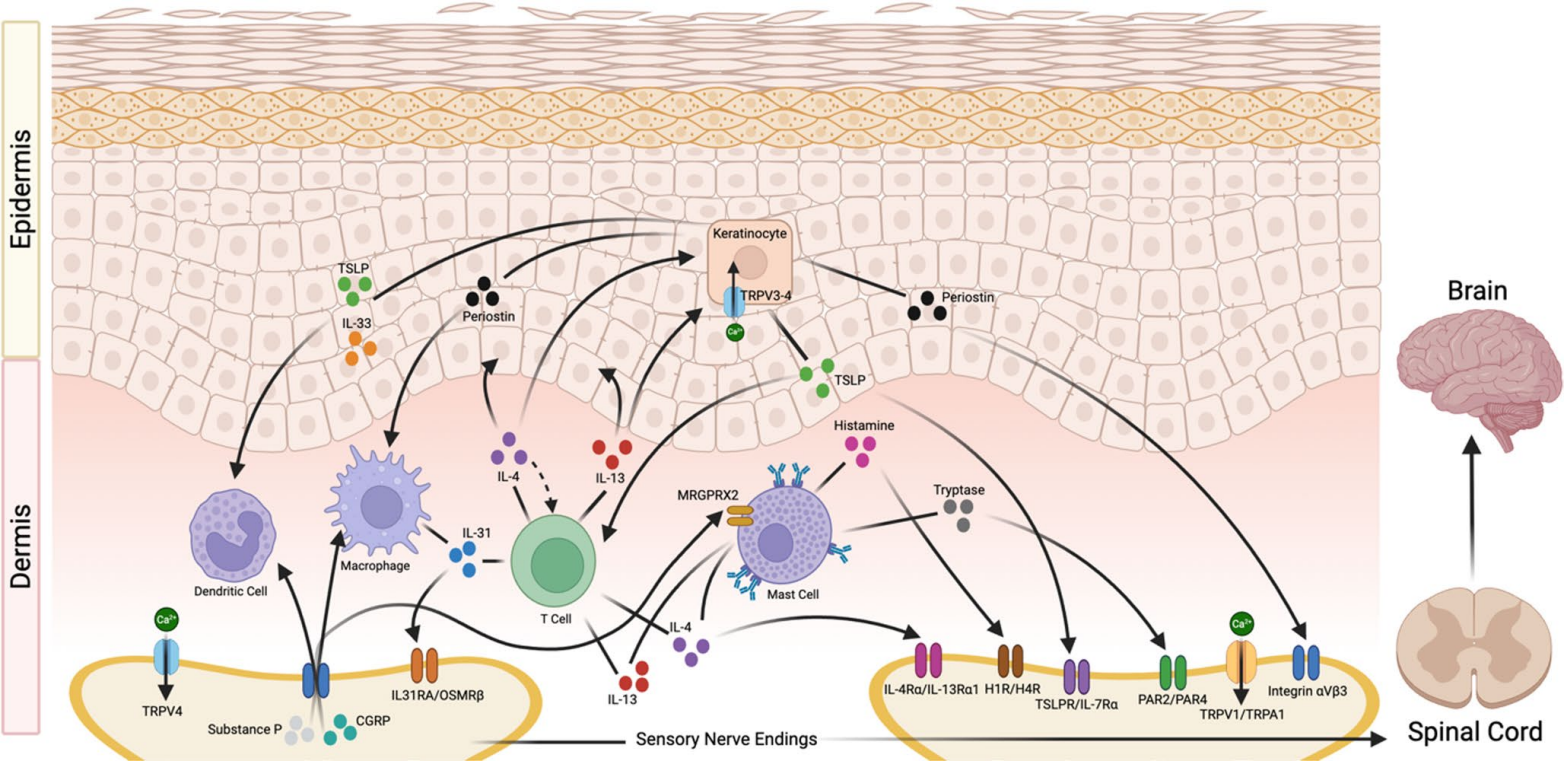
Schematic overview of the major immune cells, mediators, and cutaneous nerve ending receptors involved in the pathophysiology of chronic itch. Key immune cells include dendritic cells, M2 macrophages, Th2 cells, and mast cells, among others. Type 2 cytokines such as IL-4, IL-13, and IL-31 are released from nearly all cell types, highlighting them as major players. Keratinocyte-produced mediators include TSLP, IL-33, and periostin, which interact directly with nerve ending receptors and with immune cells to encourage production of Type 2 cytokines. The neuropeptides Substance P and CGRP are released from activated nerve endings and go on to stimulate immune cells, highlighting the bidirectional feedback loop that defines chronic itch. Figure created using BioRender.com

As outlined above, neurons and immune cells each release mediators that contribute to pruritus. However, these factors rarely act in isolation. Rather, they form a bidirectional signaling network in which cytokines sensitize nerves, neuropeptides activate immune cells, and keratinocyte-derived proteins such as periostin reinforce the cycle (Fig. 1). This molecular crosstalk explains why many chronically itchy conditions persist even when individual pathways are blocked, and why therapies targeting the shared networks between immune and neuronal signaling have shown such promise in treatment of CP [37].

Cytokine-to-neuron signaling is the first layer of this phenomenon. Type 2 cytokines such as IL-4, IL-13, and IL-31 are most commonly involved in itch, and they both promote cutaneous inflammation and directly sensitize pruriceptive neurons by enhancing TRPV1 and TRPA1 excitability [5, 6, 31]. Similarly, alarmins including TSLP and IL-33 act on TRPA1 positive nerve fibers, promoting epithelial injury and neural pathway activation [38, 39]. IL-17 and TNF, while less robust than type 2 cytokines, also induce itch via neuroimmune interactions and are key targets in conditions like psoriasis [40]. Periostin represents a particularly striking example. It is a molecule secreted downstream of IL-4/IL-13 and TSLP, where it binds integrin αVβ3 on sensory neurons, triggering itch and simultaneously inducing macrophage IL-31 release [8, 39, 41]. This dual action highlights periostin as a structural and functional link between immune and neural pathways.

Neuropeptide-to-immune signaling reflects the next layer of molecular crosstalk. Neuropeptides such as substance P and CGRP, released from activated cutaneous C-fibers, go on to activate mast cells, dendritic cells, and other immune cells, promoting further immune mediator release [42–45]. BNP is a noteworthy example produced by DRG neurons and co-expressed with IL-31 receptors. BNP induces keratinocytes to secrete pruritogenic cytokines including IL-4, IL-13, and IL-31, reinforcing the same pathways that initially stimulated its production [9, 33, 46]. BNP is therefore just one of many neuropeptides acting at the intersection of neuronal and immune pathways.

The bidirectional relationship between immune and neural mediators results in positive feedback loops that promote and sustain itch across numerous conditions, offering an explanation for why acute itch can persist and evolve into CP without appropriate intervention. Furthermore, the complexity of these networks underlying CP pathogenesis highlights why antihistamines alone are often inadequate for symptom reduction, demonstrating the importance of therapies that target shared receptors or mediators, including JAK receptors and common type 2 cytokines.

## Disease Contexts: Neuroimmune Crosstalk in Allergic and Inflammatory Skin Conditions

These molecular interactions manifest clinically across a wide range of allergic and inflammatory skin conditions. CP is one of the most burdensome symptoms reported by patients with conditions such as AD, CSU, PN, among others, resulting in significant impairments in sleep, daily functioning, and quality of life. Examining disease-specific networks reveals how shared and disease-specific mediators shape clinical presentation and therapeutic responsiveness.

### Atopic Dermatitis

AD is the most common inflammatory skin disorder, with type 2 inflammation dominating the pathogenesis. IL-4, IL-13, and IL-31, which are key mediators of AD, are type 2 cytokines produced by immune cells that exert their effects in part by directly modulating neurons in the skin [6, 44]. IL-25, IL-33, and TSLP are alarmins produced by epithelial cells that promote increased production of these type 2 cytokines [47]. IL-4, IL-13, and IL-33 have been shown to sensitize human DRG neurons to both histaminergic and non-histaminergic itch [44]. In mice models, IL-4, IL-13, and IL-31 have resulted in increased epidermal nerve growth, with IL-4 and IL-13 specifically resulting in hyperplasia, increased recruitment of Th1 and Th2 immune cells, pathogen-induced cytokine storm, and induction of genes that modulate neuroinflammation [6]. Dupilumab, an IL-4Rα blocker, reversed these effects in human models, emphasizing the importance of treatments targeting the intersection of neural and immune pathways [6]. Periostin is a downstream mediator of the type 2 inflammatory pathway that binds integrin αVβ3 on sensory neurons, triggering TRPA1 activation and resulting in itch sensation [41]. Periostin can also stimulate M2 macrophages, which induce fibrosis in AD patients, independent of IL-4 or IL-13, further characterizing itself as a prime example of a neuroimmune crosstalk mediator [41]. OX40 (CD134) is another key player located on T cells, which binds OX40L on antigen-presenting cells and cytokines (IL-4/IL-13/IL-31), thereby promoting T cell proliferation, differentiation, and survival, driving neuroimmune pruritus and contributing to the chronicity of AD [48].

### Prurigo Nodularis

PN has been increasingly recognized as a Th2-driven condition, with many pathophysiological similarities to AD, including IL-4, IL-13, and IL-31 being key players in its neuroimmune pathway [49]. Similar to AD, these immune mediators interact with sensory neurons in the skin resulting in alloknesis and hyperknesis [6, 44, 49]. IL-31 in particular is central to the neuroimmune pathway of PN, as it directly drives pruritus and neural receptor remodeling in the skin through the activation of IL-13RA/OSMRβ on DRG neurons and keratinocytes [50, 51]. OSMRβ also serves as a receptor for oncostatin M (OSM), a member of the IL-6 cytokine family, that results in skin inflammation, hyperkeratosis, and fibrosis, which are all hallmarks of PN skin lesions [50]. Dermal IL-31 expression has been found to correlate with itch intensity in PN patients [50], and drugs that block the actions of IL-31, such as nemolizumab, have been shown to rapidly decrease itch and overall disease severity [52]. Additionally, vascular endothelial growth factor (VEGF), endothelin-1, neuronal synaptogenesis signaling and neuritogenesis have also been shown to be downregulated after treatment with nemolizumab [53]. This suggests a direct role of IL-31 as a pruritogen as well as in neural remodeling, with blockage of IL-31 restoring normal cutaneous nerve density and function [53].

### Chronic Urticaria

Chronic urticaria (CU) is an allergic skin disease characterized by inducible or spontaneous appearance of transient wheals, angioedema, or both for six weeks or more [54]. The pathophysiology underlying CU is complex and can be categorized as autoallergic- relying on IgE autoantibodies to autoantigens- or autoimmune- involving IgG targeting IgE or its receptor (FcεRI) (40721160 [55]). Mast cells can be activated by either pathway, and, along with the mediators they release, they play a critical role in the neuroimmune crosstalk underlying CU pathogenesis [45]. Numerous mediators are released upon mast cell degranulation, including histamine, tryptase, NGF, leukotrienes, prostaglandins, a wide range of cytokines and chemokines, VEGF, substance P, and vasoactive intestinal peptide (VIP), among others. Neuropeptides produced by mast cells like substance P, NGF, and VIP can activate cutaneous sensory nerves through interaction with nerve endings to produce itch [45]. Notably, recent evidence suggests that neuropeptides and neurotransmitters released by mast cells and sensory nerves (including substance P, neuropeptide Y, CGRP, and VIP) likely play a role in stress-induced CU via activation of receptors other than FcεRI on mast cells [45, 56]. Mast cells express a variety of receptors that interact with immune molecules and neuropeptides, including Mrgprx2, neurokinin receptors, Tropomyosin receptor kinase (Trk)-A, CGRP 1 and 2, c-Kit, and others [45]. C-Kit activation in particular results in amplification of type 2 cytokines that directly act on and sensitize peripheral nerve endings, as well as mast cell proliferation [57]. The dual production of and response to neuropeptides by mast cells reinforces the bidirectional relationship between the immune and nervous systems in the pathogenesis of CU. The wide range of neural and immune mediators involved in the pathogenesis of CU may explain why antihistamines do not produce adequate relief in all patients, highlighting a need for continued exploration of treatments targeting mediators that facilitate crosstalk between both systems [58].

## Therapeutic Implications: from Mechanisms To Medicines

Advances in understanding the molecular and cellular drivers of pruritus have redefined the therapeutic landscape, shifting treatment away from nonspecific antipruritics toward mechanism-based interventions. The recognition that chronic itch is sustained not simply by histamine but by a network of cytokines, neuropeptides, and neuroimmune crosstalk has led to the development of biologics, small-molecule inhibitors, and experimental agents that directly target itch pathways [16].

### Cytokine Targeted Biologics

IL-4, IL-13 and IL-31 are central to pruritic dermatoses such as AD and PN. Dupilumab, an antibody against IL-4Rα, was the first biologic to transform the treatment of CP. By blocking signaling from both IL-4 and IL-13, dupilumab reduces neuronal sensitization mediated through TRPA1+ neurons and improves skin barrier function. Its efficacy extends to rapid, clinically meaningful itch reduction, highlighting the direct neuronal effects of cytokine blockade [16, 59]. More recently, selective IL-13 inhibitors tralokinumab and lebrikizumab have been introduced. Lebrikizumab has a robust anti pruritic effect, while tralokinumab has a slower antipruritic effect compared to dupilumab and lebrikizumab [60-62].

IL-31 is another potent driver of chronic itch, often described as the “itch cytokine.” Nemolizumab, an anti-IL-31 receptor A antibody has shown significant reduction in itch intensity in AD and PN. By directly blocking IL-31 induced activation of TRPV1 and TRPA1 positive neurons and its downstream effect on BNP release and keratinocyte sensitization, nemolizumab addresses both neuronal and immune arms of itch [63]. The consistent antipruritic effects across trials underscore IL-31’s centrality to CP.

OX40 and OX40L inhibitors, including amlitelimab and rocatinlimab, respectively, also offer promise as therapeutic agents via blunting of type 2 cytokine release, thereby reducing direct pruritic effects and neuroimmune modulation that drives CP in AD and potentially other conditions [64].

The upstream alarmins IL-33 and TSLP represent pipeline targets. Tezepelumab, a monoclonal antibody against TSLP approved for asthma, did not demonstrate a significant anti-pruritic effect in AD [65]. We suspect in chronic itch, blocking a single alarmin rarely silences the alarm system, as the skin compensates by rerouting signals through redundant epithelial–neural pathways. A proof-of-concept trial of etokimab (anti–IL-33) in AD also did not demonstrate anti-pruritic effects [66]. However, given their roles on amplifying inflammation and itch, blockade of these alarmins may be a plausible strategy for itch reduction in combination with other agents.

Drugs offering simultaneous blockage of multiple mediators are under development and may yield stronger anti-pruritic effects than single-target medications. Target combinations include IL-4Rα/IL-31RA, IL-13/OX40L, TSLP/IL-13, and IL-33/IL-13 [67]. Drugs with dual blockage of IL-4, IL-13 and IL-31 are still undergoing preclinical trials but are highly promising for AD and PN [68–71].

IL-17 is highly involved in psoriasis, with inhibitors of IL-17 demonstrating significant itch reduction in these patients. Ixekizumab and secukinumab are examples that have demonstrated robust rapid anti-pruritic effect in psoriatic itch, likely through direct blockage of IL-17’s effects and synergistic effects with other cytokines [72–74].

### JAK Inhibitors

JAK inhibition has produced some of the most striking antipruritic effects across chronic pruritic dermatoses. Oral small molecules upadacitinib and abrocitinib achieve rapid robust itch reduction, often within days. These reflect blockade of IL-4, IL-13, IL-31 and TSLP downstream signaling through JAK1. Topical ruxolitinib extends this benefit to localized disease with a favorable safety profile. Despite regulatory concerns over long-term systemic immunosuppression, JAK inhibitors remain an effective and potent option for dermatoses associated with CP [75–77].

### Mast Cell Modulation

Remibrutinib is a novel Bruton tyrosine kinase (BTK) inhibitor that has recently been approved for CSU. BTK is involved in FcεRI-mediated mast cell degranulation, basophil signaling, and B cell functioning, making it an ideal target for conditions driven by mast cell and basophil interactions [58]. Barzolvolimab is another emerging therapy for CSU and PN that targets c-Kit, directly reducing mast cell burden [78, 79].

### Periostin and Integrin Pathway Inhibitors

Elevated periostin levels correlate with itch severity in chronic itch disorders, making it a promising therapeutic target. Although still in the preclinical phase, inhibition of periostin or its receptor, integrin αVβ3, have shown efficacy in mouse models, reducing scratching and inflammation. Translation into clinical practice will require humanized antibodies or small molecules, but the periostin-integrin axis represents a novel therapeutic frontier [8, 80].

### MrgprX2 Inhibitors

MrgprX2 is a key receptor on mast cells and DRG sensory neurons involved in non-histaminergic itch. MrgprX2 is not simply a mast-cell receptor but a neuro-immune itch amplifier, making it a rational target for non-histaminergic pruritus [81].Its inhibition offers promise in reducing mast cell-driven non-histaminergic itch with strong preclinical data in models of CSU, PN, AD, and drug-induced pruritus [40, 82–84].

### BNP Pathway Modulation

BNP has emerged as a key neuromodulator linking neurons and immune cells. Although clinical therapies have yet to directly target BNP or its natriuretic peptide receptor A (NPRA), proof of concept studies suggest that disrupting this pathway could suppress both neuronal signaling and keratinocyte driven inflammation. Potential strategies include NPRA antagonists or antisense approaches to reduce BNP expression. Translation to the clinic will require further research, but the therapeutic rationale calls for targeted drug development [22, 46]. Despite its key role in itch neurobiology, BNP is not currently a validated biomarker for treatment selection in chronic pruritic disorders. Our preliminary studies suggest that it highly correlates with itch intensity primarly in CPUO and edlerly itch, however routine measurement is not recommended outside of research settings [9, 85].

### Neuropeptide Targeting Drugs

Attempts to block Substance P mediated itch using neurokinin 1 (NK-1) receptor antagonists have had mixed results. Early promise with agents like aprepitant and serlopitant did not translate consistently in phase 3 trials, particularly in AD and PN.

### Integrated Perspective

Cytokine directed biologics achieve substantial benefit, but residual symptoms highlight redundant signaling through neuropeptides and keratinocyte derived mediators [3, 63]. Conversely, neuropeptide antagonists alone have underperformed but may still offer value when layered onto cytokine blockage [86–88]. Looking ahead, combination therapy is likely to define the next phase of itch treatment. This may look like concomitant use of dupilumab or nemolizumab plus CGRP modulators, BNP inhibitors, or periostin inhibitors; ultimately, blocking multiple pruritogenic pathways may lead to synergistic relief. Ultimately, the therapeutic trajectory in pruritus is towards integrated, pathway-based regimens tailored to disease type [10]. The shift from non-specific immunosuppression to precision antipruritic therapy marks a major advance, yet the challenge remains to close the gap between partial relief and complete control.

## Future Directions

Despite the rapid progress in understanding and treating pruritus, several key gaps remain [1]. One important area is the incomplete characterization of periostin’s broader role in CP. While correlation with symptom severity is clear, periostin’s actions extend across structural, immune, and neuronal compartments. Whether it is primarily a marker of tissue remodeling or an active driver of neuroimmune signaling remains unresolved, and answering this will be critical for advancing periostin-directed therapeutics [8, 41]. Similarly, BNP has emerged as a compelling candidate at the neuroimmune interface, but it remains unclear whether it should be viewed primarily as a mechanistic driver, a biomarker of disease activity, or both. Clarifying BNP’s predictive value could enable stratification of patients most likely to respond to IL-31 blockade or future NPRA-targeting agents [89, 90]. Finally, while mast cell-neuron interactions are well described, the full scope of mast cell-immune-neuron loops, involving basophils, eosinophils and keratinocytes, requires further mapping to understand redundancy and hierarchy among mediators [57, 91].

The methodological landscape is evolving to address these questions. Single-cell RNA sequencing (scRNA-seq) has already revealed discrete pruriceptor populations in the DRG and subsets of cutaneous immune cells that differentially express itch-relevant receptors. Extending this to lesional versus non-lesional skin will help clarify which immune-neuronal interactions are disease specific [92, 93]. Spatial transcriptomics adds another dimension by preserving tissue architecture, allowing a three-dimensional study of neuron-immune-epidermal communication in situ [94, 95]. Combining scRNA-seq with spatial methods could generate a high-resolution atlas of pruritic skin for the first time. Beyond fMRI studies correlating itch with cortical activity, the future lies in real-time functional imaging of neuronal signaling in vivo. Genetically encoded biosensors illustrate how molecular events can be visualized within intact neural circuits in real-time [96, 97]. Adapting such biosensors to pruriceptive pathways could reveal precise dynamic signaling events in the brain, dorsal horn neurons or peripheral endings during itch, bridging wide gaps in mechanistic understanding and therapeutic evaluation.

Ultimately, these insights converge towards precision medicine approaches. Pruritic disorders are clinically heterogenous, and current therapies often succeed or fail unpredictably. Biomarker-based stratification - using serum periostin levels, BNP expression, cytokine profiles and protein activity levels could eventually help match patients to specific biologics, JAK inhibitors or future neuropeptide targeted drugs [98, 99]. The field should move beyond disease labels such as AD or PN and instead classify patients by dominant molecular drivers of itch. Such an approach would not only improve efficacy but also reduce unnecessary exposure to broad immunomodulation, setting the stage for individualized, mechanism-driven and personalized medicine.

## Conclusions

CP is one of the most common and burdensome symptoms presenting across numerous allergic and dermatologic conditions. We now understand that pruritus arises from a complex interplay between the immune system, sensory neurons, and keratinocytes. Type 2 cytokines such as IL-4, IL-13, and IL-31 are key mediators of this process, directly acting on peripheral nerves to induce itch and sensitize nerve endings. Emerging mediators, including periostin and BNP, further highlight how structural proteins and neuropeptides integrate into the neuroimmune network underlying CP. Bidirectional interactions between mast cell-produced molecules and sensory neurons, as well as keratinocyte-derived signals exemplify how these interactions evolve into positive feedback loops that transform acute itch into chronic, debilitating disease.

The success of therapeutic advances in recent years have confirmed the importance of understanding these complex underlying networks. Biologics, like dupilumab and nemolizumab, that target key immune mediators and JAK inhibitors that block critical shared pathways have provided significant reduction in itch across numerous conditions. Development of bispecific cytokine inhibitors involved in itch may further advance the treatment of CP. Furthermore, research into novel agents targeting neuropeptides and other small molecules has begun emerging and offers promise in the treatment of conditions characterized by CP. The success of these treatments highlights the importance of targeting shared pathways.

## Key References

- Butler DC, Berger T, Elmariah S, Kim B, Chisolm S, Kwatra SG, et al. Chronic Pruritus: A Review. Jama. 2024;331(24):2114-24. doi: 10.1001/jama.2024.4899.
  - This article offers an excellent summary on different types of chronc itch and its management.
- Yosipovitch G, Berger T, Fassett MS. Neuroimmune interactions in chronic itch of atopic dermatitis. J Eur Acad Dermatol Venereol. 2020;34(2):239-50. doi: 10.1111/jdv.15973.
  - This article provides a focused overview of how keratinocytes, immune cells and non-histaminergic sensory nerves interact in chronic itch, specifically in atopic dermatitis.
- Hashimoto T, Mishra SK, Olivry T, Yosipovitch G. Periostin, an Emerging Player in Itch Sensation. J Invest Dermatol. 2021;141(10):2338-43. doi: 10.1016/j.jid.2021.03.009.
  - This paper identifies periostin as a novel neuro-immune mediator of itch, showing how it directly activates sensory nerves and amplifies type 2 inflammation. Periostin is a key mediator discussed in our paper, as it is a prime example of emerging evidence on the bidirectional relationship between the nervous and immune systems in chronic itch.
- Mahmoud O, Oladipo O, Mahmoud RH, Yosipovitch G. Itch: from the skin to the brain - peripheral and central neural sensitization in chronic itch. Front Mol Neurosci. 2023;16:1272230. doi: 10.3389/fnmol.2023.1272230.
  - This review integrates evidence on how both peripheral nerve alterations and central neural sensitization contribute to the persistence of chronic itch. Neural sensitization by immune mediators is key to the neuroimmune crosstalk underlying chronic itch, making this a crucial reference to include in our paper.

## Data Availability

No datasets were generated or analysed during the current study.
